# Intra-Class Interleukin-(IL)-17 Blocker Switching: Ixekizumab as a Solution for Secukinumab Non-responders in Secondary Biologic Failure in Psoriasis

**DOI:** 10.7759/cureus.78954

**Published:** 2025-02-13

**Authors:** Abhishek De, Disha Chakraborty, SK Shahriar Ahmed

**Affiliations:** 1 Dermatology, Calcutta National Medical College and Hospital, Kolkata, IND; 2 Dermatology and Clinical Immunology, Sacramento Veterans Affairs (VA) Medical Center, Rancho Cordova, USA

**Keywords:** biologic treatment, drug resistance, il-17 blockers, ixekizumab, psoriasis treatment

## Abstract

Monoclonal antibodies, particularly interleukin-17 (IL-17) inhibitors like secukinumab and ixekizumab, have significantly advanced the treatment of severe plaque psoriasis. Clinical guidelines suggest changing biologic classes after primary treatment failure and consider switching within the same class or to a different class for secondary failures. Secondary failure was defined as the loss of response (PASI > 50% of initial value) in a patient who had previously achieved PASI 50 response at weeks 12-16.

We present a case series of five male patients with chronic plaque psoriasis, each with disease durations of 9 to 16 years. When initially treated with secukinumab (300 mg weekly for five weeks, then monthly), all patients achieved Psoriasis Area and Severity Index (PASI 90) within 4 to 12 weeks. However, they experienced secondary loss of response after 9 to 16 months, qualifying as secondary biologic failure. We then switched to ixekizumab, starting with 160 mg, followed by 80 mg every two weeks for three months, and then monthly. Significant clinical improvement was observed within two weeks of starting ixekizumab, with all patients achieving and maintaining near-clear skin (PASI 90) by six months, without significant adverse effects.

This approach is particularly relevant in regions with high latent tuberculosis prevalence where TNF-alpha inhibitors are less viable. Further research is needed to confirm these results in larger cohorts.

## Introduction

Monoclonal antibodies, particularly interleukin-17 (IL-17) inhibitors such as secukinumab and ixekizumab, have become cornerstone treatments for managing recalcitrant moderate to severe plaque psoriasis [1-3]. Clinical consensus typically advocates switching to a different class of biologics if the primary treatment fails. Yet, when facing secondary treatment failure, a switch to either a different class of biologics or another agent within the same class may be considered. Secondary failure was defined as the loss of response (PASI > 50% of initial value) in a patient who had previously achieved PASI 50 response at weeks 12-16 [4,5].

In many developing countries, such as India, the selection of biologics is limited, often leaving clinicians to choose between TNF-alpha inhibitors and IL-17 blockers. Additionally, the high prevalence of latent tuberculosis poses a relative contraindication for the use of TNF-alpha inhibitors in many plaque psoriasis patients. TNF inhibitors (TNFi) have a well-established efficacy and safety profile, making them a reliable option for psoriasis and psoriatic arthritis; however, they carry risks such as tuberculosis reactivation, loss of efficacy due to anti-drug antibodies, and potential paradoxical psoriasis. IL-17 blockers offer rapid and potent disease control with a lower TB reactivation risk but may increase susceptibility to fungal infections and exacerbate inflammatory bowel disease. JAK inhibitors, as oral agents, provide convenient administration and broad immunomodulatory effects, yet they are associated with risks such as venous thromboembolism, cardiovascular events, and serious infections, including herpes zoster. Each class has distinct benefits and limitations, necessitating individualized treatment selection based on patient-specific factors. These constraints complicate treatment options in the event of primary or secondary biologic failure [4].

This study, to our knowledge the first from Southeast Asia on this subject, highlights that switching from secukinumab to ixekizumab can effectively restore remission in patients with secondary treatment failure. In this context, we present a case series of five patients with severe plaque psoriasis who exhibited a positive response to ixekizumab following a secondary loss of response to secukinumab.

## Case presentation

We present a case series involving five male patients, with chronic plaque psoriasis, all of whom did not have psoriatic arthritis. The duration of their disease ranged from 9 to 16 years. Data were retrospectively collected from institutional medical records, including assessments of disease severity using the Psoriasis Area and Severity Index (PASI) and body surface area (BSA) involvement for each patient.

One patient had hypertriglyceridemia as a comorbidity, which was effectively managed with medication. Initially, these patients were treated with secukinumab, an anti-IL-17A antibody, administered at a dosage of 300 mg per week for five weeks, followed by a monthly dose of 300 mg. All five patients initially responded well to the treatment, achieving clear or near-clear skin (PASI 90 and above) within 4 to 12 weeks.

However, after 9 to 16 months of treatment, all patients experienced a loss of response to the standard maintenance doses of secukinumab, qualifying as secondary biologic failure. According to the Indian consensus guidelines on psoriasis management, secondary biologic failure is defined by criteria including a loss of PASI 50 response, an increase in the DLQI score by more than 5, an absolute increase in the PASI of more than 5, or an increase in the BSA of more than 10%.

We needed to transition the patients to a different biologic, and in these five cases, we chose another IL-17 blocker, ixekizumab, for various reasons. After obtaining informed consent, we initiated treatment with ixekizumab at a dose of 160 mg on the first day. This was followed by 80 mg every two weeks for three months and then 80 mg once a month thereafter.

Remarkably, all patients exhibited significant clinical improvement and a reduction in PASI scores as early as two weeks into ixekizumab treatment. The Physician Global Assessment (sPGA) scores, recorded at baseline and during each follow-up visit, also demonstrated notable improvement from the very first follow-up.

Over the six-month follow-up period, every patient achieved and sustained near-clear skin with no significant adverse effects. There was significant improvement in psoriasis severity following the switch to ixekizumab across all five patients, as reflected in their mean PASI, BSA, and sPGA scores over the 24-week period. At baseline, the mean PASI score was 34, with a BSA of 36% and an average sPGA score of 4. By 24 weeks, there was a marked reduction in disease severity, with the mean PASI dropping to 10, the BSA reducing to 7%, and the mean sPGA score improving to 1. These improvements highlight a substantial response to ixekizumab, with a decrease in PASI by an average of 24 points, BSA by 29%, and sPGA by 3 points. The most notable improvement was observed in the PASI, where the score dropped significantly from baseline levels, demonstrating the effectiveness of ixekizumab in managing moderate to severe psoriasis. Overall, the data suggests that switching to ixekizumab leads to significant clinical improvement in psoriasis severity across multiple parameters. The specifics of disease severity, duration, and scores are summarized in Table 1. 

**Table 1:** Baseline and follow-up data of the five patients who were inter-class switched to secukinumab sPGA: Physician Global Assessment; PASI: Psoriasis Area and Severity Index; BSA: Body Surface Area

Patient Number	Age	Disease Duration	Earlier Treatment	Baseline			V4=4 weeks			V5=8 weeks			V6= 12weeks			V9= 24 weeks				
				PASI	BSA	sPGA	PASI	BSA	sPGA	PASI	BSA	sPGA	PASI	BSA	sPGA	PASI	BSA		sPGA	
1	33	12	Adalimumab, Secukinumab	34	36	4	9	15	3	5	6	2	2	2	1	0		0		0
2	38	11	Secukinumab	33	28	3	12	7	2	5	2	1	3	1	1	2		1		1
3	29	9	Secukinumab	36	30	3	14	11	2	8	6	2	6	4	2	3		2		1
4	52	16	Secukinumab	30	36	3	7	5	2	6	4	1	4	2	1	1		1		0
5	42	10	Secukinumab	38	40	3	17	15	3	12	9	2	8	3	2	4		1		1

**Figure 1:**
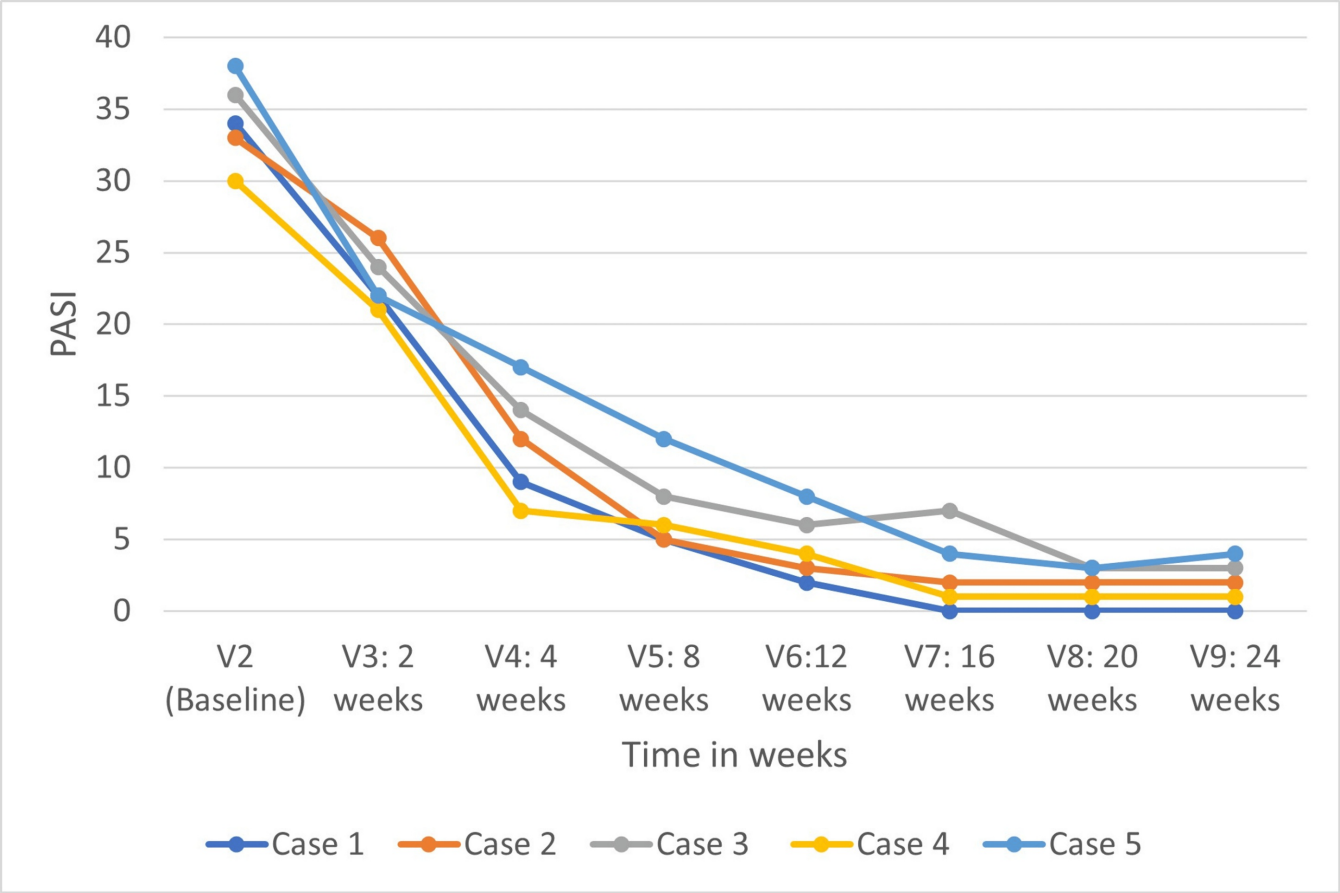
The change in the PASI score with every follow-up PASI: Psoriasis Area and Severity Index

Figure 1 shows the change in the PASI score with every follow-up. 

**Figure 2:**
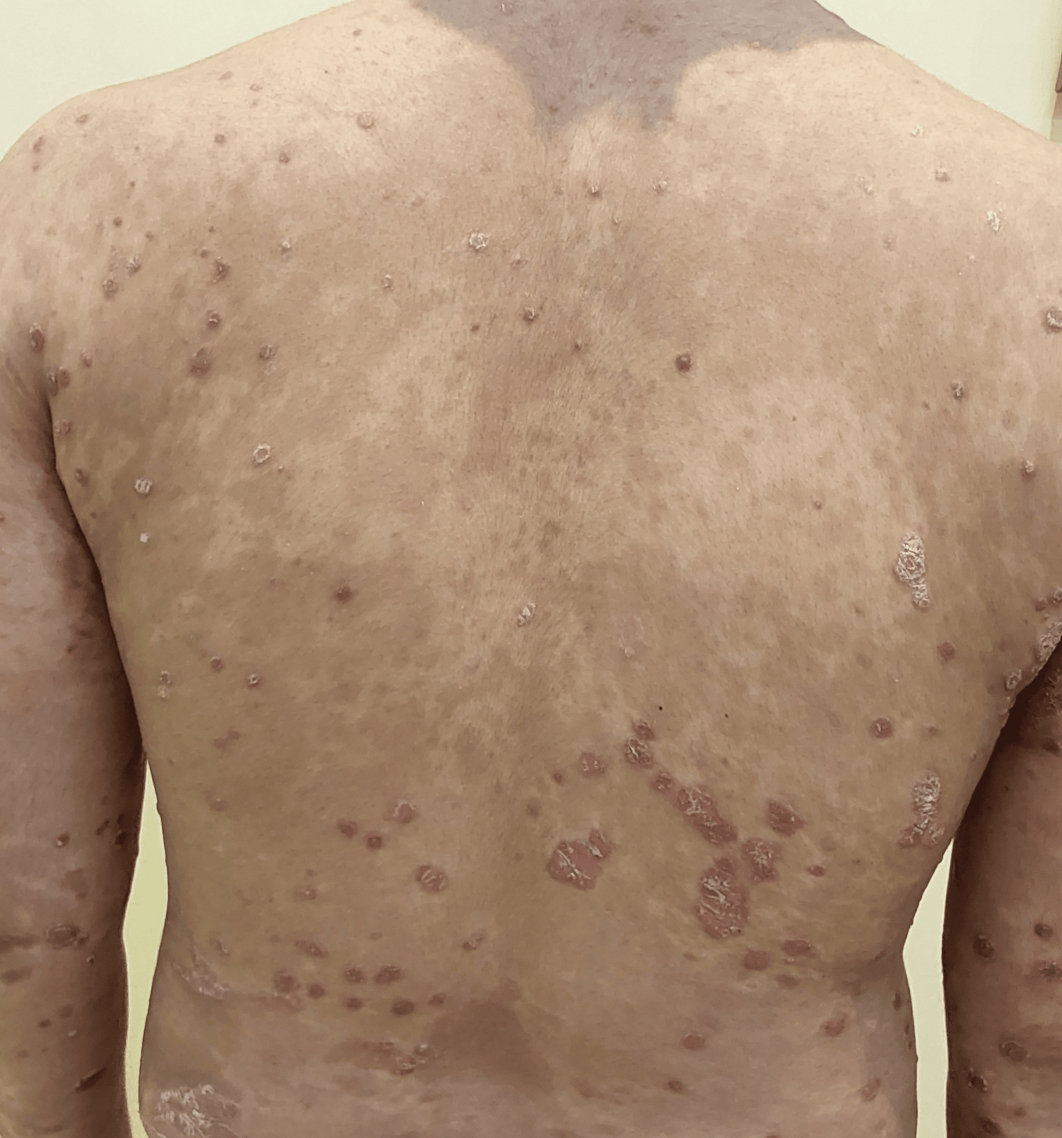
Multiple psoriatic plaques with erythema and scaling over the trunk, before starting ixekizumab

Figure 2 shows the image before starting treatment with ixekizumab in one of our patients, 38 years of age.

**Figure 3:**
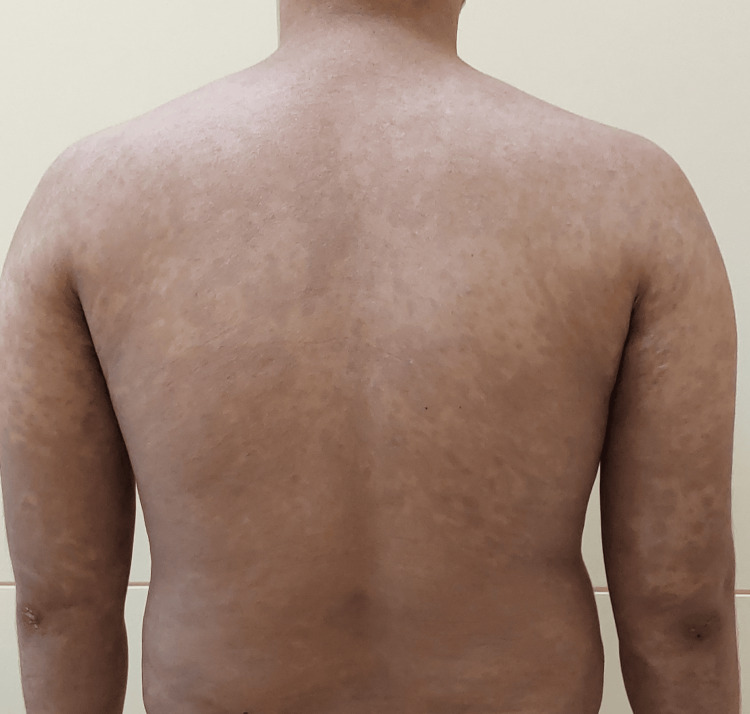
Clinical improvement in psoriasis along with PASI 100 in the same patient during the fourth month of treatment with ixekizumab PASI: Psoriasis Area and Severity Index

Figure 3 shows the image depicting clinical improvement in the same patient during the fourth month of treatment with ixekizumab.

## Discussion

With the advent of modern biologics, achieving a PASI 90 or even a PASI 100 response has become a realistic goal for many patients with moderate to severe plaque psoriasis. Yet, several challenges persist in clinical practice. Secondary resistance to biologics can develop over time, and some patients may experience a primary lack of efficacy. High-quality evidence and well-defined therapeutic algorithms for primary failure or secondary resistance to biologic therapy in psoriasis are still lacking. While professional society guidelines offer some recommendations, the ultimate treatment approach often hinges on local health policies and the clinical experience of the treating center [6-8].

Despite their similar mechanisms of action, switching between IL-17 inhibitors within the same class often proves beneficial. This advantage is likely due to the structural differences between the drugs and the specific epitope domains they target. Both secukinumab and ixekizumab bind directly to and neutralize IL-17A homodimers and IL-17A/F heterodimers. To our knowledge, this study is the first from Southeast Asia to demonstrate that switching to ixekizumab frequently leads to remission in patients who stopped responding to secukinumab. This success may be attributed to ixekizumab’s unique structure, which includes a small murine component that facilitates high-affinity binding to IL-17A [9,10]. Our findings are consistent with existing literature on this topic. 

While both drugs are IL-17A inhibitors, ixekizumab’s slightly different structure, with a higher binding affinity for IL-17A, may contribute to more effective and sustained inhibition of the IL-17A signaling pathway. This higher affinity could potentially lead to superior clinical outcomes, particularly in patients who do not respond well to secukinumab. Additionally, the pharmacokinetic profiles of the two drugs differ, with variations in half-life, peak serum concentrations, and dosing strategies, which may impact the frequency of administration and the overall consistency of treatment efficacy. These differences could also influence the therapeutic window and the ability to achieve optimal drug levels in the body over time. Furthermore, while both drugs target IL-17A, potential variations in receptor binding sites and off-target effects could play a role in their differential clinical performance. Exploring these factors would provide a deeper mechanistic understanding of why ixekizumab may be more effective in cases where secukinumab has shown suboptimal results, offering insights into personalized treatment approaches for patients with more refractory psoriasis [10].

A retrospective cohort analysis evaluated patients with moderate to severe psoriasis vulgaris who, after failing treatment with secukinumab, were switched to ixekizumab. The primary outcome was improvement in PASI scores, and the secondary outcome was the incidence of adverse events. Out of 12 patients, 91.7% achieved PASI 75, 66.7% reached PASI 90, and 8.3% attained PASI 100 by week 6. By week 12, all patients achieved PASI 75 and PASI 90, and 58.3% achieved PASI 100. No severe adverse events were reported, suggesting that ixekizumab is both effective and safe for patients who have failed other systemic or biological therapies [11]. While no significant adverse effects were reported, regular clinical assessments and laboratory monitoring were conducted to ensure patient safety. Clinical evaluations included PASI scoring, sPGA, and monitoring for signs of adverse reactions such as infections or injection-site reactions. Additionally, routine laboratory tests, including complete blood count (CBC), liver function tests (LFTs), and lipid profiles, were performed as part of standard biologic therapy monitoring. This comprehensive follow-up ensured that patients tolerated the switch to ixekizumab well, with sustained efficacy and no unexpected safety concerns.

In another multicenter, multinational retrospective study, researchers assessed treatment responses in patients who switched between IL-17 blockers. This study included patients with moderate to severe psoriasis who had not responded adequately to one IL-17 blocker (secukinumab, ixekizumab, or brodalumab) and were subsequently switched to another. The primary endpoint was achieving PASI 75 after 12 weeks of treatment. Out of 26 patients (13 male and 13 female) who underwent 29 switches, 18 switched from secukinumab to ixekizumab, seven to brodalumab, and others switched between different blockers. PASI 75 was achieved in 15 cases (52%), PASI 50 in six cases (20%), and eight cases (28%) showed no significant improvement [12]. These studies further validate our findings.

The small sample size of five patients reflects the real-world nature of this case series, which aims to highlight the clinical effectiveness of intra-class switching in a specific subset of psoriasis patients. Given the limited availability of biologics in certain regions and the relatively recent adoption of intra-class switching as a treatment strategy, large-scale data remain scarce. While our findings align with prior studies supporting the efficacy of IL-17 inhibitor switching, we acknowledge that a larger cohort would strengthen the generalizability of these results. Future prospective studies with larger sample sizes are needed to further validate this approach and refine treatment guidelines.

## Conclusions

The response of our cases indicates that switching between IL-17 blockers within the same class can be an effective strategy for patients experiencing biologic fatigue with their initial IL-17 treatment. The high incidence of LTBI restricts treatment options for psoriasis, as TNF-alpha inhibitors, unlike IL-17 blockers, are often associated with the risk of latent tuberculosis reactivation. However, larger prospective cohorts are needed to confirm these results. Intra-class switching between IL-17 inhibitors, such as from secukinumab to ixekizumab, offers significant advantages beyond mitigating tuberculosis risk. It enhances patient accessibility by allowing continued treatment within an approved and familiar biologic category, reducing administrative and insurance barriers. Additionally, it is often more cost-effective, as it aligns with existing reimbursement structures and minimizes the need for additional pre-treatment evaluations. Clinically, it maintains therapeutic continuity while leveraging structural and affinity differences between agents to restore efficacy in cases of biologic fatigue. Given these benefits, intra-class switching should be considered a valuable strategy in psoriasis management, warranting further study in broader patient populations.
